# Connectome-based prediction of future episodic memory performance for individual amnestic mild cognitive impairment patients

**DOI:** 10.1093/braincomms/fcaf033

**Published:** 2025-02-17

**Authors:** Zhengsheng Zhang, Mengxue Wang, Tong Lu, Yachen Shi, Chunming Xie, Qingguo Ren, Zan Wang

**Affiliations:** Department of Neurology, Affiliated ZhongDa Hospital, School of Medicine, Southeast University, Nanjing 210009, China; Department of Neurology, Affiliated ZhongDa Hospital, School of Medicine, Southeast University, Nanjing 210009, China; Department of Radiology, Affiliated ZhongDa Hospital, School of Medicine, Southeast University, Nanjing 210009, China; Department of Interventional Neurology, The Affiliated Wuxi People’s Hospital of Nanjing Medical University, Wuxi 214023, China; Department of Neurology, Affiliated ZhongDa Hospital, School of Medicine, Southeast University, Nanjing 210009, China; Department of Neurology, Affiliated ZhongDa Hospital, School of Medicine, Southeast University, Nanjing 210009, China; Department of Neurology, Affiliated ZhongDa Hospital, School of Medicine, Southeast University, Nanjing 210009, China

**Keywords:** Alzheimer’s disease, amnestic mild cognitive impairment, episodic memory, machine learning, magnetic resonance imaging

## Abstract

The amnestic mild cognitive impairment progression to probable Alzheimer’s disease is a continuous phenomenon. Here we conduct a cohort study and apply machine learning to generate a model of predicting episodic memory development for individual amnestic mild cognitive impairment patient that incorporates whole-brain functional connectivity. Fifty amnestic mild cognitive impairment patients completed baseline and 3-year follow-up visits including episodic memory assessments (e.g. Rey Auditory Verbal Learning Test Delayed Recall) and resting-state functional MRI scanning. Using a multivariate analytical method known as relevance vector regression, we found that the baseline whole-brain functional connectivity features failed to predict the baseline Rey Auditory Verbal Learning Test Delayed Recall scores (*r* = 0.17, *P* = 0.082). Nonetheless, the baseline whole-brain functional connectivity pattern could predict the longitudinal Rey Auditory Verbal Learning Test Delayed Recall score with statistically significant accuracy (*r* = 0.50, *P* < 0.001). The connectivity that contributed most to the prediction (i.e. the top 1% connectivity) included within-default mode connections, within-limbic connections and the connections between default mode and limbic systems. More importantly, these connections with the highest absolute contribution weight mainly displayed long anatomical distances (i.e. Euclidean distance >75 mm). These ‘neural fingerprints’ may be appropriate biomarkers for amnestic mild cognitive impairment patients to optimize individual patient management and longitudinal evaluation in a timely fashion.

## Teaser

Zhang *et al*. report that baseline whole-brain FCs within the limbic and default mode systems could effectively predict 3-year longitudinal episodic memory performance for individual aMCI patients. These ‘neural fingerprints’ may be appropriate biomarkers for aMCI patients to optimize individual patient management and longitudinal evaluation in a timely fashion.

## Graphical Abstract

**graphical abstract:**
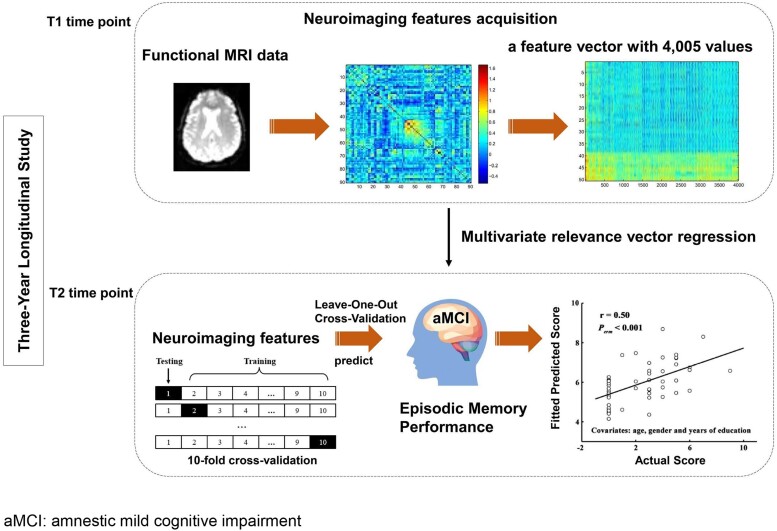

## Introduction

The main feature of Alzheimer’s disease, a progressive neurodegenerative disease, is memory loss, which is followed by issues in other areas of cognition. Alzheimer’s disease is viewed as a ‘disconnection syndrome,’ in which a progressive loss of global function and structural connections may be the cause of the subtle cognitive impairment. This is because the pathophysiology of the disease results in a progressive loss of synapsis efficacy, loss of neurons, and damage to the white matter (WM).^1-3^ Mild cognitive impairment (MCI), especially amnestic MCI (aMCI), has a relatively high risk of conversion to Alzheimer’s disease and may be an intermediate state between healthy aging and dementia.^4^ Though the annual conversion rate tends to gradually decline, approximately 10–15% of aMCI patients each year convert to Alzheimer’s disease in a very short period of time.^5^ More importantly, persons with aMCI who do not acquire Alzheimer’s disease typically either stay stable or even return to their normal state, indicating that aMCI is a widespread and extremely variable clinical dilemma. Therefore, a better understanding of the identification of individual aMCI progression could facilitate optimal decision-making for both clinicians and patients.

With the advent of non-invasive structural and functional neuroimaging techniques, connectome-based method provides an effective way to characterize Alzheimer’s disease/aMCI-related disconnection patterns, making it possible for clinicians to identify neuroimaging biomarkers of Alzheimer’s disease.^6-8^ In recent years, there has been increasing interest in multivariate pattern analysis methods (e.g. machine learning) to distinguish patients with Alzheimer’s disease/aMCI from healthy controls^9-11^ or aMCI converters from non-converters^12,13^ using different neuroimaging techniques. These studies demonstrated the feasibility of accurately identifying early Alzheimer’s disease and aMCI progression with neuroimaging biomarkers.

However, pattern classification is a dichotomous process, i.e. it assigns an individual to one category of two or more, and has often been flawed due to over-fitting.^14^ More importantly, Alzheimer’s disease pathologies present a continuous spectrum of structural and functional change. Specifically, for practical purposes, the aMCI progression is a continuous phenomenon. The range of alteration in an indicator measuring cognitive function in those aMCI patients will be multi-valued, either ordinal or continuous, rather than dichotomy.^15^ Instead of using categorical classification, it is crucial to estimate continuous clinical factors that may be related to illness stage. Particularly the combination of neuropsychological and neuroimaging data makes sense, since Alzheimer’s disease/aMCI has been associated with both cognitive and neuroimaging changes.^16^ Furthermore, after a diagnosis of aMCI, the ability to predict the future neuropsychological scores from baseline neuroimaging biomarkers is even more important, as it would be extremely valuable for both tracking progression of illness and treatment response.

Intriguingly, a number of pattern regression methods have been used for estimating Alzheimer’s disease-related clinical variables, e.g. Mini-Mental State Examination (MMSE), based on neuroimaging data.^17^ These studies demonstrated that integrating pattern regression methods and neuroimaging biomarkers might be extremely valuable for accurately estimating cognitive scores and could be helpful for tracking disease progression at the single-subject level. However, as we know, episodic memory deficit is a core symptom of aMCI^18^; the ideal neuropsychological test used for assessing episodic memory could closely reflect neuroimaging changes associated with aMCI. Rey Auditory Verbal Learning Test (AVLT) is a well-known measure of episodic memory, and in previous studies, it had a significant role in early diagnosis of Alzheimer’s disease/aMCI.^8,19,20^ Moreover, many studies have shown reliable correlation between the AVLT scores and prognosis of aMCI patients.^21,22^ Thus, it is of utmost importance to determine aMCI-relevant cognitive scores (e.g. AVLT) and then to identify neuroimaging biomarkers associated with these scores so that essential pathways from brain structure or function to episodic memory deficit can potentially be discovered.

Inspired by the above problems, in this present study, we used a multivariate relevance vector regression (RVR) method,^23^ which has been found to be an effective regression method yielding robust estimation of continuous clinical variables with reasonable diagnostic accuracy and good generalization ability,^24,25^ and a cohort design to investigate the whole-brain functional connectivity (FC) features that are predictive of the baseline and 3-year longitudinal AVLT Delayed Recall (AVLT-DR) scores of aMCI patient at the individual level. Additionally, as a complement, another two neuropsychological tests, Logical Memory Test Delayed Recall (LMT-DR) and Rey-Osterrieth Complex Figure Test Delayed Recall (CFT-DR), were additionally used to evaluate all patients’ episodic memory performance. To assess the specificity of the predictive RVR model, we further evaluated the potential of whole-brain connectivity pattern for predicting the baseline and 3-year longitudinal composite episodic memory scores (i.e. calculated by averaging the AVLT-DR, LMT-DR and CFT-DR) for individual aMCI patient. We suggest a fully automated approach to test the notion that baseline whole-brain connectivity could predict future episodic memory performance. It would be beneficial to optimize individual patient management and longitudinal evaluation in a timely manner if an independent, connectome-based technique could predict episodic memory performance instead of just classifying or predicting aMCI progression or non-progression to probable Alzheimer’s disease in a dichotomous manner.

## Materials and methods

### Participants

Patients with aMCI were recruited through a normal community health screening and newspaper advertisements at the Affiliated ZhongDa Hospital of Southeast University.^26^ Eighty-seven aMCI patients were included, who all underwent a standardized clinical interview, a neuropsychological battery assessment and multi-modal brain MRI examinations. The sample size was determined by the number of patients screened for community health and recruited through advertising during the study period. All of the participants were Chinese Han and right-handed. As described in our previous publications,^27^ all patients with aMCI met the diagnostic criteria proposed by Petersen,^28^ including (i) subjective memory impairment corroborated by the subject and an informant; (ii) objective memory performance documented by an AVLT-DR score ≤4 correct responses on 12 items when the education was ≥8 years and age was ≥51 years old (a criterion more suitable for Chinese patients for objective memory impairment,^29^ consistent with the diagnostic criteria proposed by Petersen^28^); (iii) the Mattis Dementia Rating Scale-2 (MDRS-2) score was >120, or the MMSE score was ≥24; (iv) the activities of daily living were preserved; and (v) the level was not sufficient to meet the Alzheimer’s Criteria of the National Institute of Neurological and Communicative Disorders and Stroke and the Alzheimer’s Disease and Related Disorders Association (NINCDS-ADRDA). The detailed test scores of aMCI patients enrolled in this study can be found in Supplementary File 1. Participants were excluded from the study if they had a history of neurological or psychiatric illness, major medical illness, severe visual or hearing loss, and gross structural abnormalities revealed by MR images. The Research Ethics Committee of the Affiliated ZhongDa Hospital of Southeast University approved this study, and written informed consent was obtained from all participants.

We conducted on-site follow-up on the cohort population. At follow-up (i.e. the T_2_ time point, 3 years later), both the clinical/neuropsychological assessment and parameters of MRI scanning were identical to those conducted at baseline (i.e. the T_1_ time point). To ensure data quality, all neuropsychological assessment was conducted by experienced and professional neurologists. In addition, all aMCI patients included in this study had no excessive motion artefacts (i.e. exceeding 3 mm in translational movement or 3° in rotational movement) during MRI scanning or incomplete image coverage.

### Neuropsychological examination

We used the MMSE and MDRS-2 to evaluate general cognitive function in all aMCI patients, and we used a neuropsychological battery to test episodic memory performance. The AVLT-DR, LMT-DR and CFT-DR made up this battery. As in previous studies,^30^ a composite episodic memory score was further calculated by averaging the AVLT-DR, LMT-DR and CFT-DR scores for all patients.

### Data acquisition

At Southeast University’s Affiliated ZhongDa Hospital, MRI images were collected using a 3.0 T Siemens Verio scanner with a 12-channel head coil. All patients got high-resolution T_1_-weighted and resting-state functional MRI scans. High-resolution T_1_-weighted axial images encompassing the entire brain were collected using the following 3D magnetization prepared rapid gradient echo sequence: repetition time (TR) = 1900 ms; echo time (TE) = 2.48 ms; flip angle (FA) = 9°; acquired matrix = 256 × 256; field of view (FOV) = 250 mm × 250 mm; thickness = 1.0 mm; gap = 0 mm; and number of slices = 176. Resting-state functional images were obtained for 8 min with gradient-recalled echo-planar imaging sequence: TR = 2000 ms; TE = 25 ms; FA = 90°; acquisition matrix = 64 × 64; FOV = 240 mm × 240 mm; thickness = 4.0 mm; gap = 0 mm; and number of slices = 36. Before the scan, all patients received instructions to keep their eyes closed, stay awake, calm their brains and move as barely as possible during the data collection.

### Data processing

#### Resting-state functional MRI processing

Resting-state functional data were pre-processed using the SPM8 (http://www.fil.ion.ucl.ac.uk/spm/) and DPARSF (http://www.restfmri.net/forum/dparsf) toolboxes. The first 10 functional volumes were discarded to minimize the effects of scanner stabilization and participant adaptation. The remaining images were corrected for timing differences and motion effects. The individual structural images were segmented into grey matter (GM), WM and CSF using a unified segmentation algorithm. Using the transformation parameter estimate during unified segmentation, the motion-corrected functional volumes were spatially normalized to MNI space and resampled to 3 mm isotropic voxels. Further pre-processing included linear detrending and temporal bandpass filtering (0.01–0.1 Hz), which were applied to reduce the effects of low-frequency drift and high-frequency physiological noise. We regressed out several spurious effects of nuisance covariates, including six head motion parameters, mean global signal, WM signal and CSF signal.

#### Whole-brain resting-state FC analyses

To compute resting-state FC, the automated anatomical labelling (AAL) atlas was applied to parcellate the entire GM into 90 cortical and subcortical regions. A regional mean time series was generated for each participant by averaging the time series across all voxels in this region. This resulted in 90 regional mean time series. The Pearson correlation coefficient was used to calculate the resting-state FC between each pair of areas. We obtained one symmetric correlation matrix (i.e. 90 × 90) for each aMCI patient. Then, Fisher’s *z*-transform was applied to improve the normality of the correlation coefficients. Finally, for each patient, we converted the FC matrix into a feature vector with 4005 values. The Brainnetome Atlas, which includes 246 prior cortical and subcortical regions, was applied as a validation. Previous studies have shown that the Brainnetome Atlas was a fine-grained, cross-validated atlas correlating brain anatomy with psychological and cognitive functions.^31^

### Statistical analysis

#### Individualized prediction of baseline and 3-year longitudinal AVLT-DR scores by whole-brain FC features

Based on the whole-brain FC at T_1_ time point, we applied multivariate RVR to separately predict the baseline (i.e. T_1_ time point) and 3-year longitudinal (i.e. T_2_ time point) AVLT-DR scores of unseen aMCI patients. RVR produces sparse solutions for a multivariate regression model inside a probabilistic Bayesian learning framework. Since the model weights were subjected to an explicit zero-mean Gaussian prior under this framework, the majority of the weights were set to zero, meaning that only a subset of samples—referred to as the ‘relevance vector’—were used to train the model. The weights of these samples were determined using maximum likelihood estimation. The weighted sum of the feature vectors of each ‘relevance vector’ sample was used to calculate the regression coefficients for each feature. This algorithm does not have an algorithm-specific free parameter and is computationally more efficient than other algorithms.^32^ RVR has been widely used to predict age^33^ and behaviours.^34^ We used the codes from the PRoNTo toolbox (http://www.mlnl.cs.ucl.ac.uk/pronto/) to implement RVR.

#### Prediction framework

We used leave-one-out cross-validation (LOOCV) to evaluate the models’ out-of-sample generalizability in order to measure prediction accuracy. In particular, the training set consisted of *N*-1 subjects, where *N* is the number of aMCI patients; the testing sample consisted of the remaining subjects. Each feature in the training set was linearly scaled to a range of 0–1 during the training process.^24^ This training set was then used to build an RVR prediction model. The scaling parameter obtained during the training process was used to scale the feature vectors of each testing subject during the testing process. Each patient served as the testing sample once as a result of the training and testing processes being repeated *N* times. The correlation coefficient *r* and mean absolute error (MAE) between the predicted and actual scores were used to measure the prediction accuracy.^32^ In the present investigation, we adjusted for age, sex and years of schooling when assessing the association between anticipated and real AVLT-DR scores.

#### Significance of prediction performance

To find out if the coefficient *r* and MAE were noticeably better than the outcomes predicted by chance, permutation tests were employed. In particular, the prediction process described above was used 1000 times. We permuted the behavioural scores throughout the training samples for each repetition without replacing any of them. By dividing the number of permutations that displayed a value greater than the actual value for the real sample by the entire number of permutations (i.e. 1000), the *P*-value of the mean correlation *r* was determined. Similarly, the percentage of permutations that displayed a number lower than the actual value for the actual sample was known as the *P*-value of the mean MAE.

#### Contributing features and corresponding weights

Each feature’s contribution to the model was measured by its absolute RVR weight. In the context of all other features, a higher absolute weight number denoted a larger contribution from the related feature to the prediction. Given the large number of whole-brain FC features (i.e. 4005 features), we displayed the features with the highest absolute contribution weight in the top 1%. These thresholds, although arbitrary, eliminated noise components and enabled a better visualization of the most predictive features.

We utilized the standard seven-system template image provided by Yeo *et al.*,^35^ which was initially produced via a whole-brain clustering approach that produced seven large-scale functional networks, in order to readily evaluate our findings. Network nodes from the AAL atlas were assigned to one of the seven large-scale functional modules in order to construct the a priori network modules; subcortical nodes were assigned to an eighth subcortical module. Thus, eight brain networks—the default mode, fronto-parietal, ventral attention, dorsal attention, visual, sensorimotor, limbic and subcortical systems—made up the core modular partition delineated by 90-node networks. The specific networks to which the 90 brain regions in the AAL atlas belong are presented in Supplementary Table 1.

#### Validation

We conducted two additional analyses to validate our results.

##### Validation 1

Our prediction results were validated using a 10-fold cross-validation. In particular, 10 subgroups of all patients were created, 9 of which served as the training set and the other one as the testing set. An RVR prediction model was trained on the scaled training set and used to forecast the AVLT-DR scores for the scaled testing data. Parameters obtained from training data were utilized to scale the testing data. In order to use each subset as the testing set once, this process was repeated 10 times. In the end, for each patient, the correlation coefficient *r* and MAE between the actual and projected AVLT-DR values were computed. Performance may have been impacted by data division because the entire data set was split into 10 sections at random. In order to determine the final prediction performance, the 10-fold cross-validation was carried out 100 times, and the outcomes were averaged. To determine whether the prediction performance was significant, a permutation test was run 1000 times.

##### Validation 2

We additionally included the baseline whole-brain FC characteristics into the RVR model to independently predict the baseline and 3-year longitudinal composite episodic memory scores (i.e. determined by averaging the AVLT-DR, LMT-DR and CFT-DR) in order to evaluate the specificity of the predictive RVR model. To further validate the results, we averaged the LMT-DR and CFT-DR scores of all aMCI patients and recalculated the composite situational memory score without including the AVLT-DR score for additional validation.

## Results

### Demographic and clinical characteristics

The follow-up study was performed at an average of 32 months (19–48 months). Among 87 aMCI patients, 57 completed visits at 3-year follow-up, with 16 aMCI patients converting to Alzheimer’s disease and 7 aMCI patients reverting to a normal cognitive status at follow-up. Notably, seven aMCI-to-Alzheimer’s disease patients who cannot cooperate to complete the neuropsychological battery at the 3-year follow-up were not included in this study. Therefore, the follow-up eventually comprised 50 subjects, all of whom had complete neuropsychological and MRI data. The baseline and 3-year follow-up demographic and neuropsychological examination data of the 50 aMCI patients who were finally included in the analysis are shown in Table 1. The neuropsychological examination results for executive function, information processing speed and visuospatial function are shown in Supplementary Table 2. Compared to 50 aMCI participants who completed follow-up testing, the 7 aMCI patients who could not complete follow-up testing had poorer performance in general cognitive function and episodic memory, which is presented in Supplementary Table 3.

**Table 1:** Demographic and neuropsychological examination

	aMCI patients (*N* = 50)	
	Baseline	3-year follow-up
Age (years)	68.0 ± 7.3	70.1 ± 7.3
Education (years)	11.9 ± 3.4	
Gender (male/female)	30/20	
MMSE	27.08 ± 2.03	26.30 ± 3.22
MDRS-2	133.74 ± 5.76	125.40 ± 20.65
AVLT-immediate recall	15.7 ± 3.5	14.4 ± 4.4
AVLT-recognition	19.6 ± 2.4	18.8 ± 4.2
Episodic memory	6.53 ± 2.75	6.40 ± 3.35
AVLT-DR	2.78 ± 1.54	2.46 ± 2.38
LMT-DR	3.34 ± 2.26	2.60 ± 2.02
CFT-DR	13.47 ± 6.03	14.14 ± 6.64

Data are presented as the mean ± standard deviation. aMCI, amnestic mild cognitive impairment; MMSE, Mini-Mental State Examination; MDRS-2, Mattis Dementia Rating Scale-2; AVLT-DR, Auditory Verbal Learning Test Delayed Recall; LMT, Logical Memory Test Delayed Recall; CFT-DR, Rey-Osterrieth Complex Figure Test Delayed Recall.

### Individualized prediction of baseline and 3-year longitudinal AVLT-DR scores by whole-brain FC features

**Figure 1:**
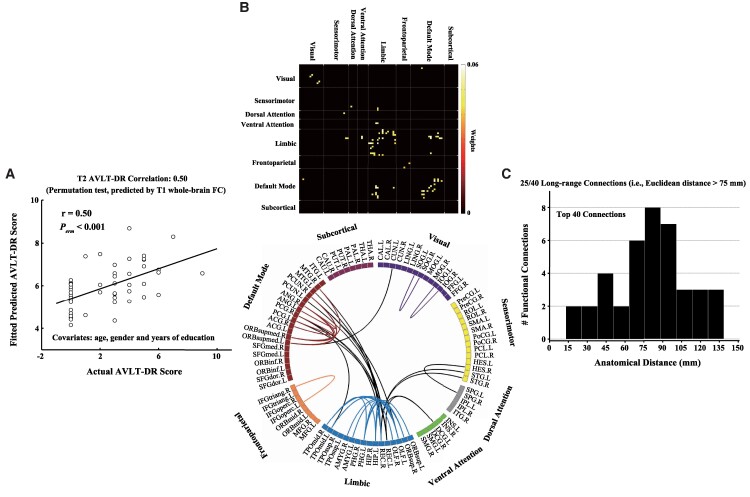
**Prediction of AVLT-DR scores for 50 aMCI patients was based on the T_1_ whole-brain FC of each individual.** Evaluated by multivariate RVR and LOOCV, the pattern of T_1_ whole-brain FC failed to predict the T_1_ AVLT-DR scores (*r* = 0.17, *P* = 0.082), but significantly predicted the T2 AVLT-DR scores (**A**; *r* = 0.50, *P* < 0.001). (**B**) By quantifying the contribution of each feature to the model based on its RVR weight absolute value, the connectivity that contributed the most to the T2 AVLT-DR score prediction (i.e. the top 1% connectivity) included within-default mode connections, within-limbic connections and the connections between default mode and limbic systems. (**C**) These connections with the highest absolute contribution weight mainly displayed long anatomical distance (25/40 long-range connections). aMCI, amnestic mild cognitive impairment; AVLT-DR, Auditory Verbal Learning Test Delayed Recall; FC, functional connectivity; T1, T_1_ time point (i.e. baseline); T2, T_2_ time point (i.e. 3-year follow-up). For the abbreviations of the brain regions, see Supplementary Table 1.

Evaluated by LOOCV, we found that the baseline whole-brain FC features failed to predict the baseline AVLT-DR scores (*r* = 0.17, *P* = 0.082). Nonetheless, the baseline whole-brain FC patterns significantly predicted the 3-year longitudinal AVLT-DR scores (Fig. 1A), with the partial correlation between actual and predicted AVLT-DR scores being *r* = 0.50 (*P* < 0.001). Furthermore, when the baseline AVLT-DR score was considered as an additional covariate, the baseline whole-brain FC features could also significantly predict the 3-year longitudinal AVLT-DR scores (partial *r* = 0.464, *P* < 0.001). Using the Brainnetome Atlas as validation, the baseline whole-brain FC patterns still significantly predicted the 3-year longitudinal AVLT-DR scores (Supplementary Fig. 2), with the partial correlation between actual and predicted AVLT-DR scores being *r* = 0.393 (*P* = 0.001). In order to further validate the reliability of the research results, we re-conducted RVR prediction by using a single well-validated feature [i.e. bilateral hippocampal volume (HV)] or combining HV and whole-brain FC features. Evaluated by LOOCV, the baseline HV could predict the 3-year AVLT-DR scores (partial *r* = 0.260, *P* = 0.004); however, a higher correlation coefficient for the combined HV and whole-brain FC features (partial *r* = 0.514, *P* < 0.001) was observed, as compared with the HV alone (Supplementary Table 4).

The connectivity that contributed the most to the 3-year follow-up AVLT-DR scores prediction (i.e. the top 1% connectivity) included within-default mode connections, within-limbic connections and the connections between default mode and limbic systems (Fig. 1B). The detailed information on FC and related brain regions is presented in Supplementary Table 5. More importantly, these connections with the highest absolute contribution weight mainly displayed long anatomical distances (i.e. Euclidean distance >75 mm) (Fig. 1C). The feature with the highest contribution weight in the top 10% (i.e. 401 functional connectivities) is shown in Supplementary Fig. 1.

### Validation results

**Figure 2:**
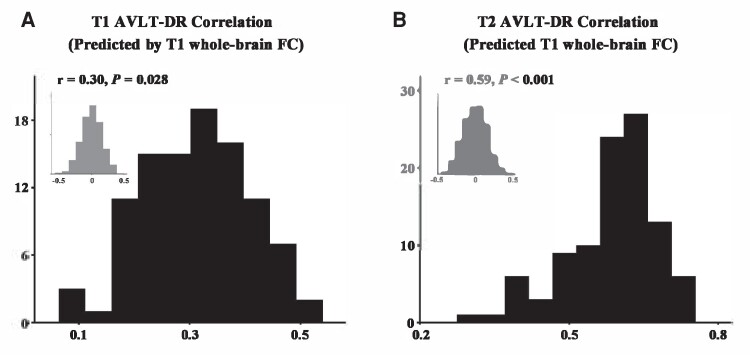
**Validation using the 10-fold cross-validation.** Repeating 10-fold cross-validation 100 times and applying permutation testing 1000 times to test the significance of predictive performance, it was found that the pattern of T_1_ whole-brain FC could predict the T1 AVLT-DR scores, but the accuracy of predicting was much more modest (**A**; *r* = 0.30, *P* = 0.028). However, the T_1_ whole-brain FC significantly predicted the T2 AVLT-DR scores (**B**; *r* = 0.59, *P* < 0.001). The distribution of permutation of the prediction *r* values is indicated by the grey bar chart, and the distribution of the prediction *r* values obtained using real scores is indicated by the black bar chart. AVLT-DR, Auditory Verbal Learning Test Delayed Recall; FC, functional connectivity; T1, T1 time point (i.e. baseline); T2, T_2_ time point (i.e. 3-year follow-up).

We first applied 10-fold cross-validation to validate our main results. The baseline whole-brain FC pattern could predict the baseline AVLT-DR scores, but the accuracy of predicting was much more modest (*r* = 0.30, *P* = 0.028; Fig. 2A). However, consistent with our main results, the baseline whole-brain FC pattern still significantly predicted the 3-year longitudinal AVLT-DR scores (*r* = 0.59, *P* < 0.001; Fig. 2B).

**Figure 3:**
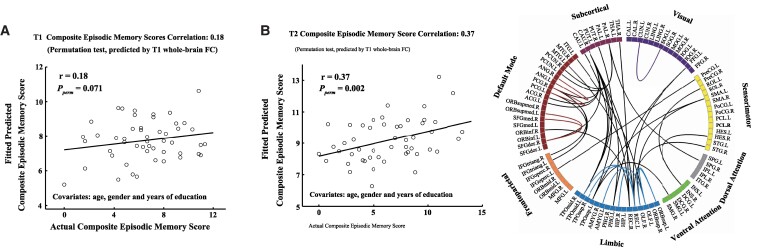
**Validation using the composite episodic memory score based on 50 aMCI patients at the follow-up (i.e. calculated by averaging the AVLT-DR, LMT-DR and CFT-DR).** Evaluated by multivariate RVR and LOOCV, the pattern of T_1_ whole-brain FC significantly predicted the T_2_ composite episodic memory scores (**B**, left panel; *r* = 0.37, *P* = 0.002) but failed to predict the T1 composite episodic memory scores (**A**; *r* = 0.18, *P* = 0.071), and the top 1% FCs for predicting T_2_ composite episodic memory scores mainly involved connections linking different functional modules (i.e. the default mode, limbic and subcortical systems) and connections within single modules (i.e. the default mode and limbic systems) (**B**, right panel). AVLT-DR, Auditory Verbal Learning Test Delayed Recall; CFT-DR, Rey-Osterrieth Complex Figure Test Delayed Recall; FC, functional connectivity; LMT-DR, Logical Memory Test Delayed Recall; T1, T_1_ time point (i.e. baseline); T2, T_2_ time point (i.e. 3-year follow-up). For the abbreviations of the brain regions, see Supplementary Table 1.

To further validate the robustness of our RVR prediction model, we calculated the average AVLT-DR, LMT-DR and CFT-DR scores as the composite episodic memory performance. The pattern of baseline whole-brain FC significantly predicted the 3-year longitudinal composite episodic memory scores (*r* = 0.37, *P* = 0.002; Fig. 3B, left panel) but failed to predict the baseline composite episodic memory scores (*r* = 0.18, *P* = 0.071; Fig. 3A); the top 1% of FCs for predicting 3-year longitudinal composite episodic memory scores mainly involved connections within single modules (i.e. the default mode and limbic systems) and connections linking different functional modules (i.e. the default mode, limbic and subcortical systems) (Fig. 3B, right panel). In addition, we recalculated the composite episodic memory score by averaging the LMT-DR and CFT-DR scores for all aMCI patients and found that baseline whole-brain FC could also predict the 3-year longitudinal composite episodic memory scores (partial *r* = 0.286, *P*_perm_ = 0.012).

Therefore, these validation results further support the model’s generalizability to predict 3-year longitudinal episodic memory performance for aMCI patients at the individual level.

## Discussion

Since the neurodegeneration of Alzheimer’s disease proceeds years before the onset of the disease and since therapeutic intervention is more effective at the early stage of the disease (e.g. the aMCI stage), there is an urgent need to (i) accurately predict the progression of the disease measured by cognitive scores, e.g. AVLT-DR, that would be more focused on functions related to aMCI and (ii) identify a small set of biomarkers most predictive of the progression. In the current study, we employed baseline whole-brain network connectivity in a machine learning framework to make continuously valued predictions on baseline and 3-year longitudinal episodic memory performance (indicated by AVLT-DR score) for aMCI patients at the individual level. We demonstrated that the whole-brain FC feature-based prediction model identified a wide range of network connections that better contributed to the prediction of 3-year longitudinal AVLT-DR scores, and these FCs mainly involved connections within and between the default mode and limbic systems. More importantly, these connections with the highest absolute contribution weight mainly displayed long anatomical distances (i.e. Euclidean distance >75 mm).

Identifying specific brain changes in the early stages before the onset of Alzheimer’s disease is crucial for clinical intervention and prevention of disease progression.^36^ Neuroimaging based on MRI is an important means of revealing changes in brain structure. In recent years, the application of artificial intelligence (AI) to analyse neuroimaging data for predicting disease prognosis and outcomes has been rapidly increasing, especially in machine learning and deep learning.^37-39^ A previous review summarizing the application of different AI in Alzheimer’s disease showed that the deep learning-based convolution neural network algorithms has the best performance in in diagnostic classification of normal aging, MCI and Alzheimer’s disease, with a weighted average accuracy of 89%, followed by support vector machine (SVM) at 86%, random forest at 84%, logistic regression at 76% and other AI methods at 81%.^40^ Deep learning models are powerful tools for solving regression or classification problems; however, they have limitations such as the need for large data sets for validation and high computational costs.^41^ The SVM algorithm is a relatively early established and also currently the most widely used machine learning method.^42^ SVM has the characteristics of high classification accuracy and strong generalization ability without the need for a large amount of data tuning.^43^ RVR is a Bayesian sparse probability model, similar to SVM, for solving regression problems. The biggest advantage of RVR is that it greatly reduces the computational complexity of kernel functions.^44^ In this study, due to the relatively small sample size and the tendency of deep learning models to overfit, we chose the RVR linear model with considerable accuracy and generalization ability. In addition, in certain neuroimaging applications, deep learning models might not be the optimal choice.^45^ By comparing the performance of deep neural network (DNN) structure and kernel regression in predicting individual phenotypes through whole-brain resting-state FC, He *et al*.^46^ found that kernel regression was competitive in all sample sizes. Considering that the predictive performance of kernel regression and DNN is comparable, and kernel regression significantly reduces computational costs, some experts believe that this classic machine learning algorithm may be more suitable for some neuroimaging applications than DNN.^46^

Previous studies have also made significant efforts in using brain imaging data to predict the progression of MCI to Alzheimer’s disease. Frizzell *et al*.’s^40^ systematic review indicates that nearly three-quarters of existing AI research focuses on the classification of Alzheimer’s disease, MCI and normal aging, while a quarter focuses on predicting the transition from MCI to Alzheimer’s disease. It will be more crucial to utilize the predictive power of AI to better identify individuals at high risk of Alzheimer’s disease, as it is conducive to providing more effective early treatment strategies. Therefore, using regression techniques instead of classification techniques may be beneficial. This study uses the RVR prediction model to predict the continuous changes in cognitive function of aMCI patients at the individual level, which is key to breaking through personalized care and treatment. To identify neuroimaging-based biomarkers for predicting cognitive scores, it has been recently advocated to push the traditional correlational analysis across all samples to the individualized prediction that naturally evaluates whether the identified neuroimaging markers can be generalized and used in practice.^47^ Given that patients with aMCI are at high risk for conversion to Alzheimer’s disease, it is of great clinical interest to be able to identify a set of biomarkers most predictive of aMCI progression. However, until now, there have been limited studies on the predictive ability of neuroimaging-based biomarkers for aMCI progression, and the accuracy of the prediction of cognitive change (i.e. MMSE changes) has been very modest. Therefore, to improve the model’s predictive accuracy, we employed the AVLT-DR test to evaluate aMCI patients’ episodic memory deficit, which is known to be a core symptom of aMCI, and combined the RVR method and longitudinal design to investigate the potential of whole-brain connectivity pattern for predicting the baseline and 3-year longitudinal AVLT-DR scores for individual aMCI patient. Our current findings indicate that the baseline whole-brain FC features cannot predict the baseline AVLT-DR scores. However, intriguingly, it could accurately predict the 3-year longitudinal AVLT-DR scores for individual aMCI patient. The ability of an independent, connectome-based technique to predict longitudinal episodic memory performance would help optimize individual patient management and longitudinal evaluation in a timely fashion. It is noteworthy that there appears to be minimal variation in episodic memory scores of participants across the two time points, but the predicted results are different. Furthermore, the trajectory of aMCI patients’ CFT-DR scores seems to be ascending in the follow-up. One possible reason is that the sample size of aMCI converters is very small, and among the 16 aMCI-to-Alzheimer’s disease patients, 7 patients who could not cooperate to complete the neuropsychological battery at follow-up were not included in further prediction analyses, while 7 subjects showed significant cognitive improvement, making the trajectory of the aMCI cohort seem cognitively stable. Compared to baseline, the distribution of neuropsychological scores on follow-up was more scattered.

The connectivity that contributed the most to the 3-year longitudinal prediction included within-default mode connections, within-limbic connections and the connections between default mode and limbic systems. More importantly, these connections with the highest absolute contribution weight mainly displayed long anatomical distances (i.e. Euclidean distance >75 mm). These findings signify the ability of connectome-based method to detect functional changes that predict longitudinal episodic memory performance for individual aMCI patient, putatively pointing to a pathological cause. Intriguingly, these findings correlate with the pathological process of Alzheimer’s disease. It is striking that early Aβ deposition occurs in a stereotypic set of heteromodal cortical regions, largely overlapping the neocortical regions of the default mode network (DMN) that are among the strongest ‘connection hubs’, whereas tau accumulation begins in deep GM structures, prominently in the transentorhinal cortex and related structures in the medial temporal lobe that are functionally connected to the DMN.^48^ Regarding Alzheimer’s disease, increasing evidence suggests that pathology may begin within key vulnerable ‘hubs’, e.g. the medial temporal lobe and default mode regions, defined as central regions within the Alzheimer’s disease pathology-targeted network architecture.^49^ These regions represent specific network ‘epicentres’ whose connectivity serves as a template for the spatial patterning of disease.^50^ Importantly, previous network findings in Alzheimer’s disease provide the strongest support for the ‘transneuronal spread model’, in which Alzheimer’s disease pathology propagates along target network connections once the disease has spread throughout the ‘hub’ regions.^51,52^ In particular, task-free fMRI studies have identified the consistent involvement of the limbic network and DMN in Alzheimer’s disease and aMCI.^53^ These networks show good topographic overlap with areas affected by pathology and neurodegeneration, that is, the hippocampus and parahippocampal gyrus, posterior cingulate and precuneus, and medial prefrontal cortex. Moreover, many previous neuroimaging studies provided consistent evidence that limbic system or DMN damage corresponds to disease progression, as measured by cross-sectional studies at different disease stages^54,55^ or longitudinal studies.^56^ Therefore, our findings provide further evidence supporting the ‘transneuronal spread model’ in Alzheimer’s disease and demonstrate that the connectome-based neuroimaging biomarkers are more beneficial for revealing aMCI progression patterns and are helpful for accurately reflecting the underlying functional changes associated with aMCI progression.

Several limitations should be considered when generalizing and extending the results in this present study. First, this study is a small sample study and the sample of aMCI converters is very small, making the trajectory of the aMCI cohort seem cognitively stable. Generalization of the current findings requires further validation using a larger independent sample and other cross-validation methods. In the future, we will expand the sample size and use public databases to further validate our results. Second, further investigations are encouraged to achieve a better and more reliable prediction performance by combining MRI-derived features (e.g. structural and functional MRI) and other modality data (e.g. APOE genotype, Alzheimer’s disease pathology derived from blood and CSF). Third, we will attempt to expand the sample size and use deep learning models with convolutional neural networks to explore better predictive performance. Finally, the study lacked amyloid imaging and histopathological data suggestive of Alzheimer’s disease pathology in aMCI patients and thus relied on clinical diagnosis only. Nonetheless, only aMCI patients who were deemed to have a high risk of developing Alzheimer’s disease dementia were included in the current study at baseline. More notably, aMCI patients had significantly smaller cortical thickness in the hippocampus and parahippocampal gyrus,^27^ decreased plasma Aβ40 and Aβ42 levels,^57^ and greater plasma neurofilament light levels^57^ compared to healthy controls, as demonstrated in our earlier investigations using the same data set. As a result, it is possible that the aMCI patients included in this study were almost uniform. A large independent aMCI sample diagnosed by amyloid imaging and histopathological data is warranted in further study to validate our preliminary findings.

## Conclusion

We demonstrated that baseline whole-brain FCs within the limbic and default mode systems could effectively predict 3-year longitudinal episodic memory performance for individual aMCI patient. These ‘neural fingerprints’ may be appropriate biomarkers for aMCI patients to optimize individual patient management and longitudinal evaluation in a timely fashion.

## Supplementary Material

fcaf033_Supplementary_Data

## Data Availability

The data that support the findings of this study are available from the corresponding author upon reasonable request.
