# Supplementary material for: Connectome-based prediction of future episodic memory performance for individual amnestic mild cognitive impairment patients
Source: Brain Commun. 2025 Feb 17;7(1):fcaf033. doi: 10.1093/braincomms/fcaf033 (PMC11831076; doi:10.1093/braincomms/fcaf033)
Supplement: fcaf033_Supplementary_Data [file fcaf033_supplementary_data.zip › Supplementary_Material.pdf]

Connectome-Based Prediction of Future Episodic Memory Performance for Individual Amnestic Mild Cognitive Impairment Patient

Supplementary AVLT score prediction equation

After evaluating the model with leave-one-out cross-validation, we constructed a model using all participants. As RVR is a linear prediction model, therefore the equation of the prediction function is y = w * x + b. The variable 'w_Brain.xlsx' stores the weights ('w') of all features and 'w_alpha_end.xlsx' stores the constant term ('b'). Using these data, the AVLT scores can be predicted for any unseen participant with whole-brain FC feature values.

The top 10% of connectivity with the highest contribution to predicting the 3-year AVLT-DR scores

We reanalyzed and displayed the feature with the highest contribution weight in the top 10% (i.e., 401 functional connectivities). As shown in the Fig S1, the connectivity that contributed the most to the 3-year AVLT-DR scores mainly included within-default mode connections, within-limbic connections, and connections between default mode and limbic systems; and connections between default-mode/limbic and other systems (e.g.,

dorsal/ventral attention and subcortical) also made a contribution to this 3-year AVLT-DR scores' prediction.

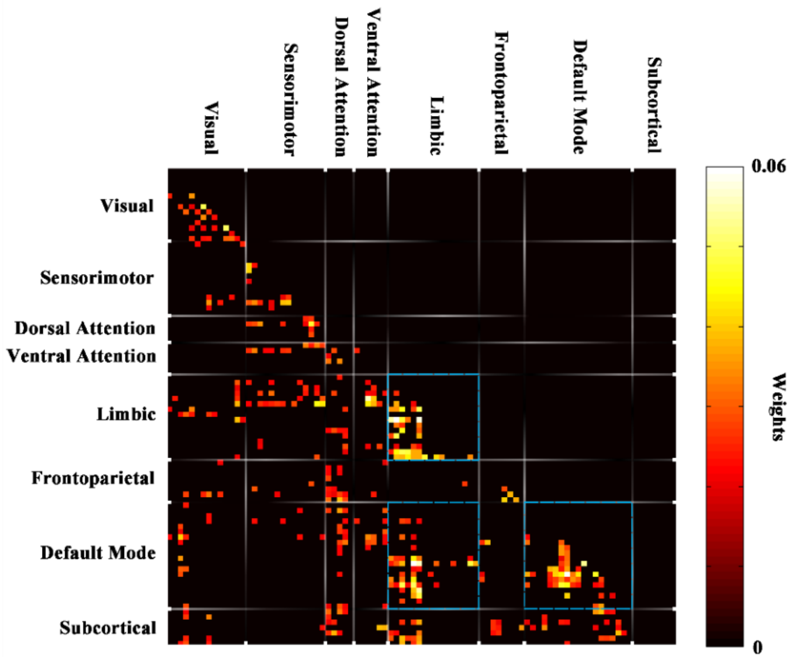

Figure S1. The connectivity that contributed the most to the T2 AVLT-DR scores prediction (i.e., the top 10% connectivity).

T2 AVLT-DR Correlation :0.393

(Permutation test, n = 1000; predicted by T1 whole-brain FC defined by the *Brainnectome atlas*)

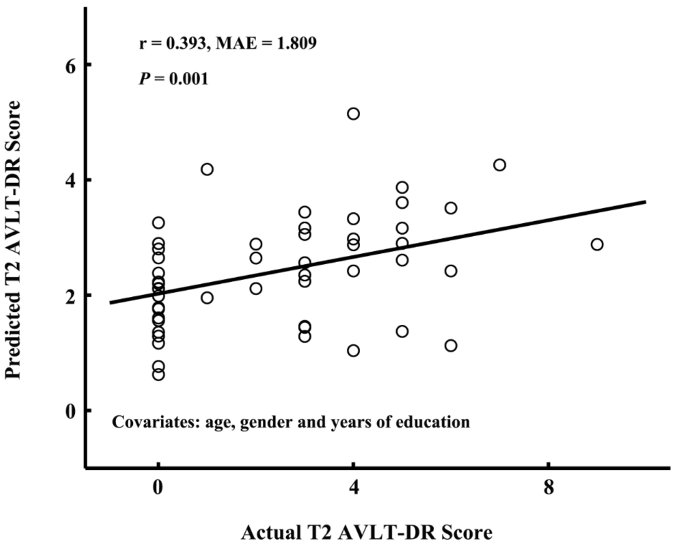

Figure S2. The pattern of T1 whole-brain FC based on the Brainnectome atlas significantly predicted the T2 AVLT-DR scores.

SUPPLEMENTARY Table S1. Cortical and subcortical regions of interest (ROIs) included in AAL atlas.

Labels	Regions	Abbreviation	Network
1	Precentral gyrus_left	PreCG.L	Sensorimotor
2	Precentral gyrus_right	PreCG.R	Sensorimotor
3	Superior frontal gyrus, dorsolateral_left	SFGdor.L	Default Mode
4	Superior frontal gyrus, dorsolateral_right	SFGdor.R	Default Mode
5	Superior frontal gyrus, orbital part_left	ORBsup.L	Limbic
6	Superior frontal gyrus, orbital part_right	ORBsup.R	Limbic
7	Middle frontal gyrus_left	MFG.L	Frontoparietal
8	Middle frontal gyrus_right	MFG.R	Frontoparietal
9	Middle frontal gyrus, orbital part_left	ORBmid.L	Frontoparietal
10	Middle frontal gyrus, orbital part_right	ORBmid.R	Frontoparietal
11	Inferior frontal gyrus, opercular part_left	IFGoperc.L	Frontoparietal
12	Inferior frontal gyrus, opercular part_right	IFGoperc.R	Frontoparietal
13	Inferior frontal gyrus, triangular part_left	IFGtriang.L	Frontoparietal
14	Inferior frontal gyrus, triangular part_right	IFGtriang.R	Frontoparietal
15	Inferior frontal gyrus, orbital part_left	ORBinf.L	Default Mode
16	Inferior frontal gyrus, orbital part_right	ORBinf.R	Default Mode
17	Rolandic operculum_left	ROL.L	Sensorimotor
18	Rolandic operculum_right	ROL.R	Sensorimotor
19	Supplemental motor area_left	SMA.L	Sensorimotor
20	Supplemental motor area_right	SMA.R	Sensorimotor
21	Olfactory cortex_left	OLF.L	Limbic
22	Olfactory cortex_right	OLF.R	Limbic
23	Superior frontal gyrus, medial_left	SFGmed.L	Default Mode

24	Superior frontal gyrus, medial_right	SFGmed.R	Default Mode
25	Superior frontal gyrus, medial orbital_left	ORBsupmed.L	Default Mode
26	Superior frontal gyrus, medial orbital_right	ORBsupmed.R	Default Mode
27	Gyrus rectus_left	REC.L	Limbic
28	Gyrus rectus_right	REC.R	Limbic
29	Insula_left	INS.L	Ventral Attention
30	Insula_right	INS.R	Ventral Attention
31	Anterior cingulate and paracingulate gyri_left	ACG.L	Default Mode
32	Anterior cingulate and paracingulate gyri_right	ACG.R	Default Mode
33	Median cingulate and paracingulate gyri_left	DCG.L	Ventral Attention
34	Median cingulate and paracingulate gyri _right	DCG.R	Ventral Attention
35	Posterior cingulate gyrus_left	PCG.L	Default Mode
36	Posterior cingulate gyrus_right	PCG.R	Default Mode
37	Hippocampus_left	HIP.L	Limbic
38	Hippocampus_right	HIP.R	Limbic
39	Parahippocampal gyrus_left	PHG.L	Limbic
40	Parahippocampal gyrus_right	PHG.R	Limbic
41	Amygdala_left	AMYG.L	Limbic
42	Amygdala_right	AMYG.R	Limbic
43	Calcarine fissure and surrounding cortex_left	CAL.L	Visual
44	Calcarine fissure and surrounding cortex_right	CAL.R	Visual
45	Cuneus_left	CUN.L	Visual
46	Cuneus_right	CUN.R	Visual
47	Lingual gyrus_left	LING.L	Visual
48	Lingual gyrus_right	LING.R	Visual

49	Superior occipital gyrus_left	SOG.L	Visual
50	Superior occipital gyrus_right	SOG.R	Visual
51	Middle occipital gyrus_left	MOG.L	Visual
52	Middle occipital gyrus_right	MOG.R	Visual
53	Inferior occipital gyrus_left	IOG.L	Visual
54	Inferior occipital gyrus_right	IOG.R	Visual
55	Fusiform gyrus_left	FFG.L	Visual
56	Fusiform gyrus_right	FFG.R	Visual
57	Postcentral gyrus_left	PoCG.L	Sensorimotor
58	Postcentral gyrus_right	PoCG.R	Sensorimotor
59	Superior parietal gyrus_left	SPG.L	Dorsal Attention
60	Superior parietal gyrus_right	SPG.R	Dorsal Attention
61	Inferior parietal, but supramarginal and angular gyri_left	IPL.L	Dorsal Attention
62	Inferior parietal, but supramarginal and angular gyri_right	IPL.R	Dorsal Attention
63	Supramarginal gyrus_left	SMG.L	Ventral Attention
64	Supramarginal gyrus_right	SMG.R	Ventral Attention
65	Angular gyrus_left	ANG.L	Default Mode
66	Angular gyrus_right	ANG.R	Default Mode
67	Precuneus_left	PCUN.L	Default Mode
68	Precuneus_right	PCUN.R	Default Mode
69	Paracentral lobule_left	PCL.L	Sensorimotor
70	Paracentral lobule_right	PCL.R	Sensorimotor
71	Caudate nucleus_left	CAU.L	Subcortical
72	Caudate nucleus_right	CAU.R	Subcortical
73	Lenticular nucleus, putamen_left	PUT.L	Subcortical
74	Lenticular nucleus, putamen_right	PUT.R	Subcortical

75	Lenticular nucleus, pallidium_left	PAL.L	Subcortical
76	Lenticular nucleus, pallidium_right	PAL.R	Subcortical
77	Thalamus_left	THA.L	Subcortical
78	Thalamus_right	THA.R	Subcortical
79	Heschl gyrus_left	HES.L	Sensorimotor
80	Heschl gyrus_right	HES.R	Sensorimotor
81	Superior temporal gyrus_left	STG.L	Sensorimotor
82	Superior temporal gyrus_right	STG.R	Sensorimotor
83	Temporal pole: superior temporal gyrus_left	TPOsup.L	Limbic
84	Temporal pole: superior temporal gyrus_right	TPOsup.R	Limbic
85	Middle temporal gyrus_left	MTG.L	Default Mode
86	Middle temporal gyrus_right	MTG.R	Default Mode
87	Temporal pole: middle temporal gyrus_left	TPOmid.L	Limbic
88	Temporal pole: middle temporal gyrus_right	TPOmid.R	Limbic
89	Inferior temporal gyrus_left	ITG.L	Default Mode
90	Inferior temporal gyrus_right	ITG.R	Dorsal Attention

Abbreviations: AAL, automated anatomical labeling.

SUPPLEMENTARY Table S2. The Neuropsychological examination of the aMCI group.

	aMCI patients (N = 50)		
	**Baseline**	**3-year Follow-up**	**P value**
**Executive Function**			
VFT-animals	17.14±4.1	16.8±5.19	0.952
DST-background	4.4±1.37	4.1±1.4	0.475
TMT-B	235.72±97.06	242.20±101.39	0.657
Stroop C	100.76±32.15	98.58±36.08	0.684
Similarity	17.12±3.96	14.20±4.01	0.002
**Information Processing Speed**			
DSST	30.74±9.43	28.86±9.23	0.551
TMT-A	79.34±25.42	88.76±27.57	0.105
Stroop A	31.68±7.14	33.04±8.36	0.746
Stroop B	46.90±12.54	49.06±14.66	0.671
**Visuospatial Function**			
CDT	8.26±1.54	8.18±1.89	0.784
CFT	33.84±2.3	31.74±6.66	0.086

Data are presented as the mean ± standard deviation.

Abbreviations: aMCI, amnestic mild cognitive impairment; CDT, clock drawing test; CFT, Rey-Osterrieth complex figure test; DST, digit span test; DSST, digit symbol substitution test; Similarity, semantic similarity test; Stroop A, B, and C, Stroop color-word test A, B and C; TMT-A and B, trail making tests A and B; VFT, verbal fluency test.

SUPPLEMENTARY Table S3. Comparison of the baseline demographic and clinical characteristics of seven aMCI patients who could not complete follow-up testing and others who could complete follow-up.

	7 aMCI that did not complete the follow-up testing	aMCI patients (N = 50)	*P* value
Age (years)	71.71±7.45	68.0±7.3	0.209[b]
Education (years)	10.57±3.15	11.9±3.4	0.319[b]
Gender (male/female)	2/5	30/20	0.221[a]
MMSE	23.57±7.23	27.08±2.03	<0.001[b]
MDRS-2	123.86±5.98	133.74±5.76	<0.001[b]
AVLT-immediate recall	9.71±4.89	15.7±3.5	<0.001[b]
AVLT-recognition	15±3.06	19.6±2.4	<0.001[b]
Episodic Memory	2.86±4.68	6.53±2.75	0.001[b]
AVLT-DR	0.86±1.21	2.78±1.54	0.003[b]
LMT-DR	1.07±2.09	3.34±2.26	0.001[b]
CFT-DR	6.64±12.26	13.47±6.03	0.019[b]

Data are presented as the mean ± standard deviation.

[a] chi-square test

[b] two sample t-test

Individualized prediction of 3-year longitudinal AVLT-DR scores by bilateral hippocampal volume

In order to further validate the reliability of the research results, we re-conducted RVR prediction by using a single well validated feature (i.e., bilateral hippocampal volume [HV]) or combining HV and whole-brain functional connectivity (FC) features. As shown in the table below, evaluated by leave-one-out cross-validation, the baseline HV could predict the 3-year AVLT-DR scores (partial $r = 0.260$, $P = 0.004$); however, a higher correlation coefficient for the combined HV and whole-brain FC features (partial $r = 0.514$, $P < 0.001$) was observed, as compared with the HV alone.

SUPPLEMENTARY Table S4. RVR prediction based on HV or the combination of HV and whole-brain FC features.

**Feature**	**Prediction *r***	***P* value**
Combined	0.514	< 0.001
Whole-brain FC	0.501	< 0.001
Bilateral HV	0.260	0.004

Abbreviations: FC, functional connectivity; HV, hippocampal volume.

SUPPLEMENTARY Table S5. The connectivity that contributed the most to the T2 AVLT-DR scores prediction (i.e., the top 1% connectivity).

Contributing Features (functional connectivity)		Network Modules	Functional Connectivity (*Z* values)	Corresponding Weights
**Brain Region 1**	**Brain Region 2**			
ORBsupmed.R	ANG.R	Default Mode	0.705	0.063
REC.L	PCG.R	Limbic - Default Mode	0.026	0.063
ORBsup.R	PHG.L	Limbic	0.682	0.060
REC.R	PHG.L	Limbic	0.885	0.058
REC.R	PCG.R	Limbic - Default Mode	1.007	0.056
DCG.L	REC.L	Ventral Attention - Limbic	0.320	0.056
ORBsup.L	PHG.L	Limbic	0.426	0.054
REC.R	MTG.L	Limbic - Default Mode	0.308	0.054
PCG.L	PCG.R	Default Mode	0.454	0.053
REC.L	PCUN.R	Limbic - Default Mode	1.167	0.050
IOG.L	IOG.R	Visual	0.280	0.050
OLF.L	PCUN.R	Limbic - Default Mode	0.249	0.050

OLF.L	TPOmid.L	Limbic	0.538	0.049
REC.R	HIP.L	Limbic	0.761	0.049
ORBsup.	AMYG.R	Limbic	0.640	0.049
SOG.L	SOG.R	Visual	0.681	0.049
TPOmid.L	PCG.R	Limbic - Default Mode	-0.073	0.048
REC.R	MTG.R	Limbic - Default Mode	0.228	0.048
OLF.R	TPOmid.L	Limbic	0.483	0.048
ORBsupmed.L	PCUN.R	Default Mode	1.096	0.048
HIP.L	TPOmid.R	Limbic	0.274	0.047
HES.R	SPG.R	Sensorimotor - Dorsal Attention	1.001	0.047
OLF.L	PHG.L	Limbic	0.815	0.047
ANG.L	ANG.R	Default Mode	0.106	0.046
OLF.L	HIP.L	Limbic	0.979	0.045
OLF.L	AMYG.L	Limbic	0.491	0.045
STG.L	REC.R	Sensorimotor - Limbic	0.864	0.045
ORBsupmed.R	PCUN.R	Default Mode	0.304	0.045
ORBsup.R	TPOmid.L	Limbic	0.681	0.045
OLF.R	TPOmid.R	Limbic	0.279	0.044

REC.L	PCUN.L	Limbic - Default Mode	0.770	0.044
ORBsupmed.L	ANG.R	Default Mode	0.313	0.044
REC.L	PCG.L	Limbic - Default Mode	0.268	0.044
DCG.L	REC.R	Ventral Attention - Limbic	0.186	0.043
STG.R	REC.R	Sensorimotor - Limbic	0.710	0.043
ACG.R	ANG.R	Default Mode	0.420	0.043
PHG.L	TPOmid.R	Limbic	0.237	0.042
IFGoperc.L	IFGtriag.R	Frontoparietal	0.278	0.041
CUN.L	ORBsupmed.L	Visual - Default Mode	1.402	0.041
ORBsupmed.R	MTG.R	Default Mode	-0.078	0.041

Abbreviations: AVLT-DR, auditory verbal learning test delayed recall; T2, T2 time point (i.e., 3-year follow-up). For the abbreviations of the brain regions, see *Supplementary Table S1*.
